# A new bats echolocation-based algorithm for single objective optimisation

**DOI:** 10.1007/s12065-016-0134-5

**Published:** 2016-02-18

**Authors:** Nafrizuan Mat Yahya, M. Osman Tokhi, Hyreil Anuar Kasdirin

**Affiliations:** Department of Automatic Control and Systems Engineering, University of Sheffield, Sheffield, UK

**Keywords:** Optimisation, Bats echolocation, Reciprocal altruism, Bats sonar algorithm, Adaptive bats sonar algorithm

## Abstract

Bats sonar algorithm (BSA) as a swarm intelligence approach utilises the concept of echolocation of bats to find prey. However, the algorithm is unable to achieve good precision and fast convergence rate to the optimum solution. With this in mind, an adaptive bats sonar algorithm is introduced with new paradigms of real bats echolocation behaviour. The performance of the algorithm is validated through rigorous tests with several single objective optimisation benchmark test functions. The obtained results show that the proposed scheme outperforms the BSA in terms of accuracy and convergence speed and can be efficiently employed to solve engineering problems.

## Introduction

In general, optimisation is the process of obtaining either the best minimum or best maximum result under specific circumstances [16, 29]. Most of the engineering problems in, for example, engineering design, manufacturing processes and control are solved by employing optimisation approaches [16]. Over the past four decades, researchers have developed various types of algorithms for solving a range of engineering optimisation problems [12]. Among these is the evolutionary and metaheuristic algorithm [25] which is based on combination of rules and randomness, simulating natural phenomena such as animal behaviours or processes of biological evolution [1, 12]. Swarm intelligence has been categorised under evolutionary algorithms. Swarm intelligence techniques are developed based upon modelling the collective behaviour of social group of living species, for instance; colony of ants, bacteria, bees, bats, birds and fish [1, 8]. In general, swarms have self-organisation and decentralised control features and all the swarm follows the same system where a population of swarm cooperates and interacts with each other in the group and the environment under certain rules during foraging or socialising purpose [8, 25].

Nowadays, swarm intelligence raised a lot of attention from the research community. There are many swarm intelligence algorithms that have developed recently to solve single objective optimisation problems. Yang [26] presented a firefly algorithm (FA) that was encouraged from the unique pattern of flashing light by a swarm of fireflies. The FA idealised from three rules; all fireflies are unisex, attractiveness is proportional to their brightness and objective function landscape determines the brightness. Yang [26] compared the performance of FA with GA and PSO on ten single objective optimisation benchmark test functions. The results indicated FA outperformed both of the algorithms regarding the efficiency and success rate. In the same year, [28] developed a cuckoo search (CS) algorithm that was based on the obligate brood parasitic behaviour of some cuckoo species. This algorithm is also integrated with the Lévy flight behaviour of some birds and fruit flies. The CS algorithm operates based on three rules inspired by cuckoo breeding behaviour. The rules are: each cuckoo lay one egg in a random nest at a time, the best nest with the highest quality of eggs will bring forward to next generations and fixed number of available host nests. The CS algorithm has been verified and compared with GA and PSO on ten single objective optimisation benchmark test functions. The simulation results showed that CS performed better as compared to both established algorithms especially for multi modal objective functions [28].

In 2012, a new swarm intelligence algorithm, the krill herd (KH) algorithm was proposed by [7]. The KH algorithm is based on the simulation of the herding behaviour of krill individuals. The KH algorithm sets the minimum distances and highest density of krill herd from food as the objective function. Besides, KH algorithm also has taken movement induced by the presence of other individuals, foraging activity and random diffusion as three main factors to determine the time-dependent position of each krill. The KH algorithm has been compared with other eight existed algorithms to solve twenty single objective optimisation benchmark test functions. The result validated a better performance of the KH algorithm to solve the benchmark test functions as well as outperform other established algorithms [7]. Then, [18] developed a hybrid algorithm of ant colony optimisation and firefly algorithm (ACO-FA) algorithm for solving single objective optimisation problems. The ACO-FA combined the advantages of both swarm intelligence algorithms where ant colony works as a global searcher and firefly colony works as a local searcher. Rizk-Allah et al. [18] performed the ACO-FA algorithm on a set of fifteen single objective optimisation benchmark test functions. The simulation results suggested that the ACO-FA algorithm demonstrated better performance for searching the global optimum solution as compared to other prominent algorithms.

Next, [4] developed an algorithm inspired by bird mating strategy during mating season. The bird mating optimiser (BMO) algorithm is aimed to solve the single objective optimisation problems. In BMO algorithm, the population is called *society* and in each *society* member is called a *bird* that represented a feasible solution. There are five groups of *bird*s in the society based on the real birds mating system. The groups are parthenogenetic, polyandrous, monogamous, polygynous and promiscuous. The BMO algorithm was tested on three categories of single objective optimisation benchmark test functions. The categories are unimodal functions, multimodal functions and low-dimensional multimodal functions. The simulation results showed a better performance of BMO algorithm to provide a good balance between global and local search effectively as compared to other existing algorithms [4]. Recently, [13] proposed a social network-based swarm optimisation algorithm (SNSO) targeted for solving single objective optimisation problems. The SNSO algorithm adopted a social network evolution model of the swarm to improve the search performance of a swarm. The SNSO introduced a dynamical population topology, extended neighbourhood structure and divided the individuals into two groups based on their fitness. Results from computer simulation on twelve single objective optimisation benchmark test functions were validated that SNSO achieved better performance as compared to seven others distinguished population-based algorithms [13].

Nevertheless, swarm intelligence algorithms based on bats also appeared in the literature. Among significance bats-based algorithm were bat algorithm (BA) by [27] and bats sonar algorithm (BSA) by [21]. Both algorithms are inspired from echolocation of a colony of the bats. This paper introduces an adaptive version of the algorithm proposed by [21]. The modifications introduced are based on the nature of echolocation of bats so as to address the shortcomings of the original algorithm mentioned above. The paper is organized as follows. The unique echolocation behaviour of bats is first described in Sect. 2. The BA by [27] and BSA by [21] are described in Sect. 3. The adaptive bats sonar algorithm (ABSA) is presented in Sect. 4. The performance of ABSA and BSA reflecting the number of bats and number of iterations is discussed in Sect. 5. Comparative assessment of ABSA with the BSA and BA is presented with several single objective optimisation benchmark test functions in Sect. 6, and the paper is concluded in Sect. 7.

## Bats echolocation

As one of the diverse and most extraordinary mammalian order, bats have more than 900 species distributed all around the world [3, 23]. According to [17] and [22], bats generally live in a large colony with 700–1000 individuals under sharing roosts.

The social calls and echolocation calls are two types of acoustic communication used by a colony of bats [22]. A colony of bats is able to construct good communication and sharing information between each other about roost site or foraging area [3]. According to [3], there are four basic information transfer mechanisms in a colony of bats:

1. Intentional signalling: in the form of mating calls, territorial calls, alarm calls or food calls (advertisement of food and also to attract bats into foraging groups as they leave their cave roosts).
2. Local enhancement: involves unintentionally directing another bat to a specific part of the habitat.
3. Social facilitation: an increase in individual foraging success brought about by group foraging behaviour.
4. Imitative learning: bats can learn foraging techniques from other bats.

**Fig. 1:**
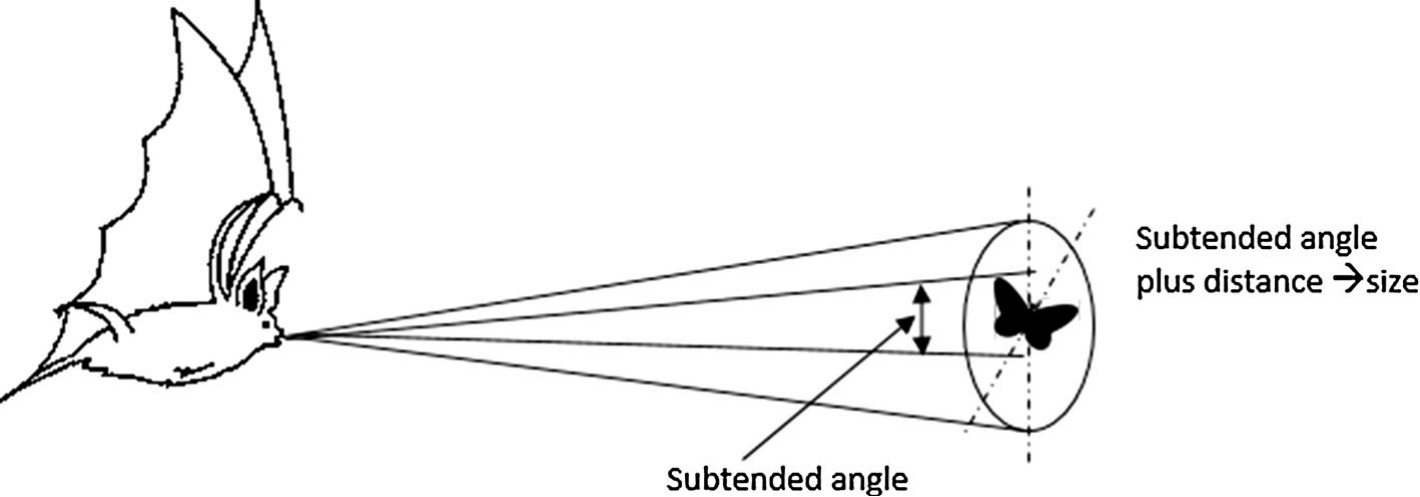
Sonar signal of a bat [20]

The term ’echolocation’ was described by Griffin in 1944 as the ability of bat to produce sound with echo beyond the frequency range of human hearing and use for general orientation and finding prey [2]. In echolocation, a bat emits ultrasonic pulses in short burst through mouth [3] as shown in Fig. 1. The sound reflects back as echoes bump into an object in the bat’s path. Altringham et al. [3] and [20] agreed that by computing the time of reflection of modulates echoes, the bat is able to recognise the object and its distance.

The echolocation process of bats involves three phases to search and capture prey: search phase, approach phase and terminal phase [3]. During the search phase, the bat will start to hunt for prey by emitting the pulse at low rate with frequency around 10Hz. Then, the pulses have to get shorter as the time between the pulse and echo is decreased in order to avoid overlap when the bat spots and gets nearer to the specific prey during the approach phase [3, 20]. In this phase too, pulse emission rate gets steadily increased up to 200 per second since the bat keeps updating the position of the prey [3, 20]. In the terminal phase, the frequency of emitted pulses upsurges more than 200 Hz as the pulse emission rate also starts to accelerate at only fraction of millisecond long just before the prey is netted [3].

The concept of reciprocal altruism of food sharing also exists during the echolocation process in a colony of bats [3, 5, 24]. This social behaviour is based on bats returning favours to their mutual benefit [3]. For instance, vampire bats species share the blood-meals between the individuals in a colony as a response to balance energy budget amongst in a colony [3, 5]. The bats successfully establish an individual survivorship in a colony after implementing this behaviour such that the fitness of the recipient is allocated comparatively to a non-recipient [24].

## Bat algorithm and bats sonar algorithm

### Bat algorithm

Bat algorithm (BA) by [27] is developed based on echolocation behaviour of bat species to find their prey. Bat form three-dimensional of surrounding by integrating the production of the sound pulse and echo recognition time difference, the variant intensity of the sound pulse and the time delay between ears of the bat. In a such way, the bat can identify the type, moving speed, distance and orientation of the prey.

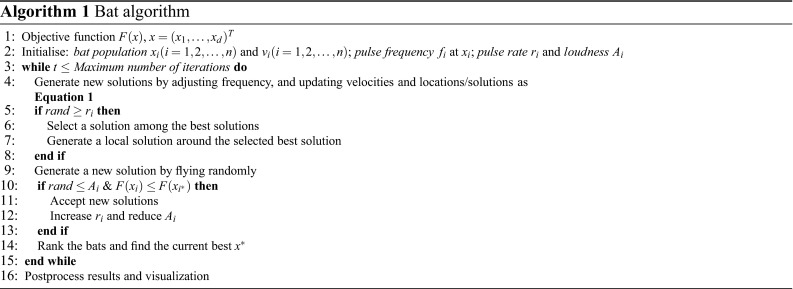



To simplify, the algorithm was developed based on the ideal rules which are [27]:

1. All bats use echolocation to detect distance and differentiate between food, prey and obstacles.
2. Bats fly randomly with velocity $v_{i}$ at position $x_{i}$ by fixed frequency $f_{min}$ with varying wavelength $\lambda$ and loudness $A_{0}$ to search for prey.
3. Bats can spontaneously adjust the wavelength or frequency and the rate of sound pulse emission $r \in [0,1]$ depending on the proximity of their target.
4. Loudness of emitted sound pulse assumed varies from a large positive $A_{0}$ to a minimum constant value $A_{min}$.
5. No ray tracing is used in estimating the time delay and the three dimensional topography.
6. Wavelength ($\lambda$) and frequency (*f*) of emitted sound pulse are related due to the fact $\lambda f$ is constant, so a range of $[f_{min},f_{max}]$ is corresponds to a range of $[\lambda _{min},\lambda _{max}]$.
7. Wavelength (or frequency) range can be adjusted and the largest wavelength (or frequency) should be selected to suit the size of the domain of the considered problem, and then toning down to smaller ranges.
8. Assume $f \in [0,1]$ even though higher frequencies have short wavelengths and travel a shorter distance.
9. The rate of sound pulse emission was in the range [0, 1] where 0 means no pulses at all and 1 means the maximum rate of pulse emission.

The developed BA is pictured in pseudo code as in Algorithm 1. In this algorithm, [27] updated the velocity $v_{i}$ and position $x_{i}$ of bats’ movement in a *d*-dimensional search space as Eq. 1:

1 $$\begin{aligned} \begin{aligned} f_{i}&=f_{min}+(f_{max}-f_{min})\beta \\ v_{i}^t&=v_{i}^{t-1}+(x_{i}^t - x_{*})f_{i}\\ x_{i}^t&=x_{i}^{t-1}+v_{i}^t \end{aligned} \end{aligned}$$

where

- $x_{i}^t$ is new solution of position at time step *t*
- $v_{i}^t$ is new solution of velocity at time step *t*
- $\beta \in [0,1]$ is random value
- $x_{*}$ is the recent global best solution which is derived
-    after examining every solutions among *n* bats

To update the velocity of the new solution, either $f_{i}$ or $\lambda _{i}$ could be used while fixing the other factor as velocity increment as a product of $\lambda _{i}f_{i}$. The value of $f_{i}$ (or $\lambda _{i}$) is important to control the pace and range of the movement of the bats [27]. In other hand, values of $f_{max}$ and $f_{mim}$ have been fixed as $f_{min}=0$ and $f_{max}=100$ where each bat has its random frequency that allocate uniformly around the fixed above values. However, the values have relied on the problem domain size.

According to [27], a new position for every bat is produced using random walk after a solution is chosen among the current best positions as Eq. 2:

2 $$\begin{aligned} x_{new}=x_{old} + \varepsilon A^t \end{aligned}$$

where

- $\varepsilon \in [-1,1]$ is a random number
- $A^t=\left\langle A_{i}^t\right\rangle$ is the average loudness of all the bats at this time step.

Usually, when a bat approaches its prey, the loudness ($A_{i}$) will decrease but the rate of pulse emission $r_{i}$ increases. Initially, every bat owns dissimilar random loudness values and pulse emission rate. So, as iteration proceeds and the new solutions are better, these two parameters have to be updated respectively [27]. For example, this algorithm used $A_{0}=1$ and assuming $A_{min}=0$ where a bat is moving to the prey and momentarily stop producing any sound. In contrasts, the algorithm used $r_{0}=0$ and assuming $r_{max}=1$ where a bat increases its pulse emission rate once approaching the prey. So Eq. 3 is derived as:

3 $$\begin{aligned} A_{i}^{t+1}= & {} \alpha A_{i}^t \nonumber \\ r_{i}^{t+1}= & {} r_{i}^0 [1-exp(-\gamma t)] \end{aligned}$$

where

$$\begin{aligned} \alpha =\gamma =0.9 \end{aligned}$$

The BA method has been implemented on various test functions including Rosebrock’s function, the egg crate function, De Jong’s standard sphere function, Ackley’s function and Michalewicz’s test function. In all implementation, the numbers of bats (*n*) used were 25 to 50. The BA has been compared with standard GA and PSO algorithms in terms of the number of function evaluations for a fixed tolerance to show the better performance of BA. The fixed tolerance was set up at $\varepsilon \le 10^{-5}$ and ran for 100 iterations. According to the results, the BA is more accurate and efficient compared to GA and PSO algorithms.

### Bats sonar algorithm

The bats sonar algorithm or (BSA) by [21] is developed based on echolocation process of a colony of bats to find food or prey. During echolocation, bats can figure out the size, distance, velocity, azimuth and elevation of the target by using the sonar. The BSA models the principles of bat sonar used in echolocation to search the optimum solution for a specific problem. Each point (prey location detected) in the search space (specific confined area) represents one possible solution. A bat is labelled as one sonar unit.

Tawfeeq [21] starts the BSA by setting the *solution range* or the minimum and maximum values of the search space. Then, the *beam length* (*L*) is initialise as in Eq. 4:

4 $$\begin{aligned} L\le Rand \times \frac{\text {Solution}\,\text{range}}{2} \end{aligned}$$

At every iteration, [21] has selected random *starting angle* ($\theta _{m}$) as well as used one of two *angle between beams*; either $Fixed_{\theta }$ which randomly select a small fixed value $\theta$ between any two successive beams or $Rand_{\theta }$ which randomly select a different angle $\theta _{i}$ between any two successive beams.

Tawfeeq [21] mentioned that the sonar unit will transmit a number of sonar signals or *number of beams* (*N*) with *L* length from the designated starting point ($pos_{s}$) to several different directions. The $pos_{s}$ also evaluates the value of *starting point fitness function* ($F_{s}$). Every beam’s *end point position* ($pos_{i}$) is calculated as Eq. 5:

5 $$\begin{aligned} pos_{i}=pos_{s}+L \cos (\theta _{m}+(i-1))\theta \end{aligned}$$

Then, the $pos_{i}$ is evaluated for the value of *end point fitness function* ($F_{i}$). The value of $F_{i}$ and $F_{s}$ is compared with each other to determine the optimum one. If the optimum value belongs to one of the $F_{i}$, the sonar unit (the bat) will fly to its $pos_{i}$ and set the point as a new $pos_{s}$. Then, the new number of *N* beams will be transmitted from this point to search for better optimum solution. Otherwise, the bat will stay at the original $pos_{s}$ and retransmit the *N* beams to different direction. The process keeps on repeating and stops once the algorithm arrives at the maximum iteration (or finds the best fitness function). Algorithm 2 pictured the pseudo code of the developed BSA. The BSA is a parallel search type where several solutions are checked simultaneously. Over iterations, only the best fitness of each bat will survive and the best fitness among the best bats’ fitness will become the global best fitness [21]. Using this way, the proposed algorithm will converge to the optimum best fitness faster.

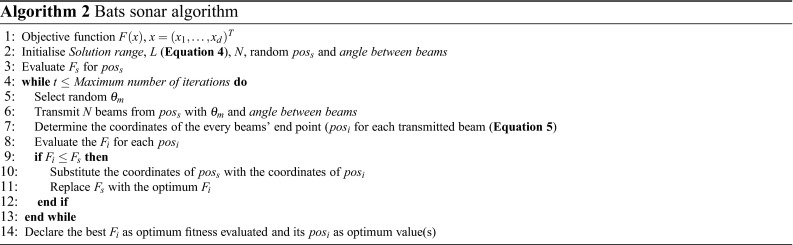



This algorithm started with the single sonar unit (SSU). Then, the development of the proposed algorithm was expanded to another two efficient search approaches [21]. If only SSU approach was being used, the result is not guaranteed to obtain the global best fitness even it converges toward the minimum or maximum fitness especially in complex problems with wide state space. The two approaches mentioned were multi sonar search unit (MSU) and single sonar unit with a momentum (SSM). In multi sonar unit (MSU), a colony of bats will search for the optimum solution(s) at the same time where each bat (sonar unit) will be assigned with different starting point in the same search space. Meanwhile, a single sonar unit with a momentum (SSM) introduced a *momentum term* ($\mu$) attached to the length of the transmitted beams so that new beam length becomes as Eq. 6:

6 $$\begin{aligned} L_{new} =L_{old}(1\pm \mu ) \end{aligned}$$

where

$$\begin{aligned} 0<\mu <1 \end{aligned}$$

Nonetheless, both approaches still use SSU algorithm as the algorithm framework [21].

To demonstrate the performance of the developed algorithm, the BSA were tested and evaluated on different types of fitness functions [21]. The initial parameters set to be the same for all tests included $N = 5,\, Fixed_{\theta } = \pi /12$ and 100 maximum iterations. The performances of BSA were measured by the degree on how much the obtained solution meets the goal where the goal is assumed to be equal or approximately equal to the optimum solution. Comparison of the developed algorithm with a genetic algorithm on the same fitness functions has been made. The comparison involves the value of obtained fitness functions and the execution time required to attain each function. The results concluded the bats sonar algorithm performed reasonable efficiency to achieve all the optimum values.

As a matter of fact, the BSA is only tested on single objective optimisation problems. Till today, no extended version of the algorithm, neither the modification to the original algorithm, hybridisation with another technique nor application to any optimisation area was reported.

### Several problems existed in bats sonar algorithm

There are some drawbacks associated with the BSA introduced by [21]. There is no communication between bats in a colony to exchange information on current location or the best locations of individual bats during echolocation process. This lack makes the algorithm as a parallel search technique. The number of bats used in the algorithm is too small and not portraying the normal population size of a colony of bats (normally in the order of hundreds) when searching for prey. The small population does not make the exploration and exploitation for the best fitness value optimum in the search space.

Furthermore, it is highly possible that the *N* beams will be transmitted in the same direction and location. This problem happens because the main transmit angle is fixed as well as roughly set up of random values of the angle between beams. These drawbacks will lead to premature convergence as the algorithm will diverge from the global best position but converge to local best location. Thus, the algorithm does not perform well to achieve the best accuracy while maintaining good precision and fast convergence to the optimum solution.

BSA also fail to capitalise several good characteristics in the real behaviour of bats echolocation into the algorithm. This failure makes BSA unable to operate like the real process of echolocation of a colony of bats. BSA is not considered the issues such as there are three phases lead to catching the prey, as well as the reciprocal altruism model of food sharing between a colony of bats.

## Adaptive bats sonar algorithm

An adaptive bats sonar algorithm (ABSA) is proposed as an improved version of original bats sonar algorithm (BSA) by [21]. The purpose of ABSA is to solve single objective optimisation problems. Overall, the ABSA has more steps than the original bats sonar algorithm BSA introduced by [21].

However, the *number of iterations* (*MaxIter*) or generations used in ABSA is kept at 100, it is the same number used in the original algorithm by [21]. 100 generations are favourably enough for the bats to explore fully the *d* numbers of search space *dimension* (*Dim*) for the best prey or *global best fitness*, ($F_{GB}$). The chosen value is in line with maximum *MaxIter* which was used in the particle swarm optimisation (PSO) algorithm when the algorithm was first introduced by [10].

Inspired by a description of the number of bats in a colony by biologists, the *number of bats* (*Bats*) or population in ABSA was selected in the range 700–1000 bats. The new population was higher by only three bats that was used in the BSA [21]. By having a larger number of bats, a discovery of the $F_{GB}$ value becomes more resourceful such that there will be a pool of solutions (prey) that can be evaluated to obtain the best ones.

In the original BSA by [21], the *beam length* (*L*) is initialised as a random value but not more than half of the *solution range* ($SS_{size}$). The solution range is the value between the *upper search space* ($SS_{Max}$) limit and the *lower search space* ($SS_{Min}$) limit as Eq. 7:

7 $$\begin{aligned} SS_{size}=SS_{Max}-SS_{Min} \end{aligned}$$

The value of *L* is constant throughout the iterations. This fixation pushes every bat to search in larger perimeter each time without the opportunity to diversify the search tactic during iterations and thus may miss the $F_{GB}$ that may be near to them. To resolve such weaknesses, the ABSA sets the *L* in relation to $SS_{size}$ as Eq. 8:

8 $$\begin{aligned} L\le Rand \times \left( \frac{SS_{size}}{10\,\%\times Bats}\right) \end{aligned}$$

The solution range is divided into micron scale, such as 10 % of the overall population of bats in the search space. The percentage is marked as possible search space size of each bat to emit sound without colliding with one another. The value of *L* is different for every iteration. A *momentum term* ($\mu$) is used in ABSA as Eq. 9:

9 $$\begin{aligned} L_{new}=L_{old}(1\pm \mu ) \end{aligned}$$

where

$$\begin{aligned} 0<\mu <1 \end{aligned}$$

The above has been introduced by [21] to control the risk of convergence to a local optimum.

Tawfeeq [21] has fixed the *number of beams* (*NBeam*) emitted by each bat at each iteration to five. This value is too small and obviously only a part of the bat’s surrounding is covered by the pulses and thus the exploitation of *local best fitness* ($F_{LB}$) and exploration of $F_{GB}$ do not occur. Such a small value also does not illustrate the real echolocation of bats. Altringham et al. [3] and Suga [20] have reported that the pulse emission rate grows bit by bit up to 200 per second as the bat keeps updating the location of the object until it catches the prey. This phenomenon is incorporated into the ABSA approach as *beam number increment* (*BNI*).

The *BNI* is defined in terms of the *maximum number of beams* ($NBeam_{Max}$) and *minimum number of beams* ($NBeam_{Min}$) as Eq. 10:

10 $$\begin{aligned} BNI=\left( \frac{NBeam_{Max}-NBeam_{Min}}{MaxIter}\right) \times iter \end{aligned}$$

where

$$\begin{aligned} NBeam_{Max}&= 200 \\ NBeam_{Min}&= 20 \end{aligned}$$

Thus, *NBeam* is defined as Eq. 11:

11 $$\begin{aligned} NBeam={NBeam_{Min}}+BNI \end{aligned}$$

The *BNI* method mimics the original pulse rate emitted by the bat as it increases gradually toward the end of the search. As a result, *BNI* will provide a balance between global exploration and local exploitation thus requiring less iteration on average to find a sufficiently optimum solution.

**Fig. 2:**
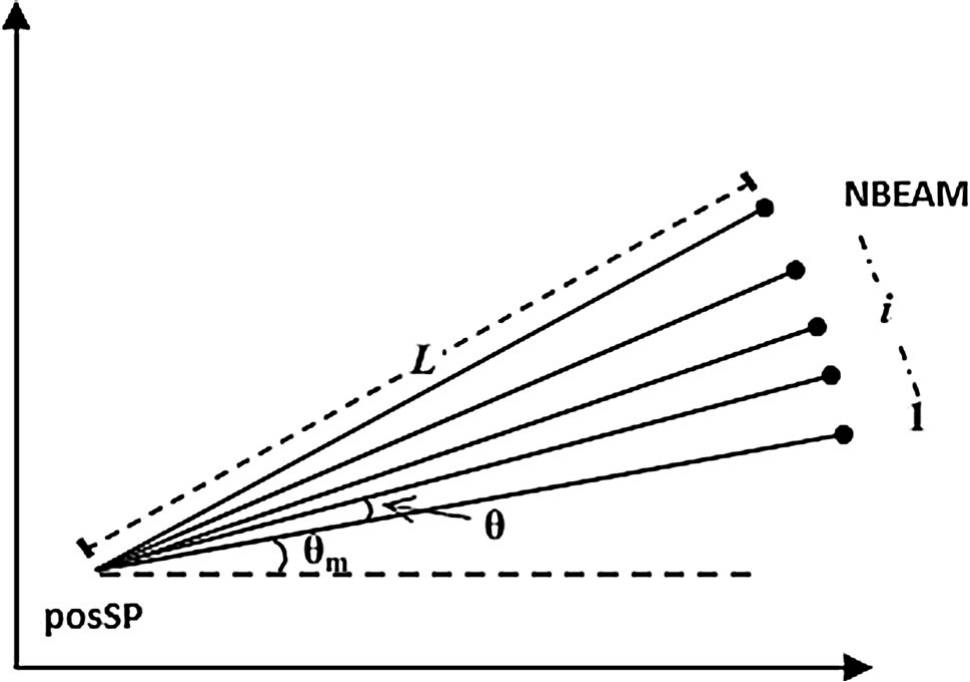
Single batch of beams transmitted by a bat [21]

Each *NBeam* with *L* is emitted from the *starting position* ($pos_{SP}$) with specific angle location. Tawfeeq [21] has selected random *starting angle* ($\theta _{m}$) at every iteration, see Fig. 2. For the angle between beams, the algorithm’s initiator uses one of the following:

1. $Fixed_{\theta }$: randomly select a small fixed value $\theta$ between any two successive beams.
2. $Rand_{\theta }$: randomly select a different angle $\theta _{i}$ between any two successive beams.

In this manner, the beam transmitted will sweep at random angles at each iteration. However, the bats fail to verify that the sounds have spread to every corner of their surroundings and it is possible that the beam will be transmitted to the same point(s) at different iterations. As a consequence, the algorithm will get trapped at $F_{LB}$ and will be unable to find the $F_{GB}$. To resolve this problem, ABSA limits the first beam to have $\theta _{m}$ not more than $45^{\circ }$ from horizontal axis and the *angle between beams* ($\theta _{i}$) is set as Eq. 12 follows:

12 $$\begin{aligned} \theta _{i}=\frac{(2\pi -\theta _{m})}{NBeam} \end{aligned}$$

where

$$\begin{aligned} \theta _{m}=rand\le 0.7854 \end{aligned}$$

By setting $\theta _{i}$ as such, the beams will sweep at random $360^{\circ }$ around the bats through iterations in such a way that the searching process will neither be too aggressive (overlay a circle) nor too slow (underlay a circle).

The *end point position* ($pos_{i}$) for each transmitted beam in ABSA is calculated the same way as in [21] as Eq. 13:

13 $$\begin{aligned} pos_{i}=pos_{SP}+L\cos [\theta _{m}+(i-1)\theta ] \end{aligned}$$

where

$$\begin{aligned} i=1,\ldots ,N \end{aligned}$$

The BSA declares a fitness at that position as the optimum fitness function once the algorithm has reached either the end of a fixed number of iterations or all solutions have converged to the same value [21]. The one level declaration of best solution is consistent with the nature of the algorithm as a parallel search method where the algorithm checks for the solutions at once. Nonetheless, the level of best fitness solution found in the algorithm has been raised up to four stages in the developed ABSA. The duo are mentioned before; $F_{LB}$ and $F_{GB}$, while another two levels are *starting position fitness* ($F_{SP}$) and *regional best fitness* ($F_{RB}$).

During the first iteration of ABSA, $pos_{SP}$ of $F_{SP}$ for each bat to transmit the *NBeam* is randomly selected within the designated search space. Next, the $pos_{i}$ for each transmitted beam from $pos_{SP}$ of each bat will be evaluate to produce *end point fitness* ($F_{i}$) where the best $F_{i}$ is declare as $F_{LB}$ and its position as *local best position* ($pos_{LB}$) of each bat. Later, the $F_{SP}$ and $F_{LB}$ of each bat is compared where the best will be $F_{RB}$ and its position as *regional best position* ($pos_{RB}$). Finally, the best of the $F_{RB}$ will be declared as $F_{GB}$ and its position as *global best position* ($pos_{GB}$). According to [6], there are three levels of best solution found by the algorithm in PSO. The levels are *personal best* (*pb*) which is the best solution for every particle, *local best* (*lb*) which is the neighbourhoods best solution and *global best* (*gb*) is the global best solution of among the *pb*. These three levels are similar to $F_{LB}$, $F_{RB}$ and $F_{GB}$ of ABSA respectively.

In PSO, the *lb* improve the overall performance of algorithm where the individual *lb* influenced the performance of immediate neighbours [9, 11]. Ultimately, the neighbourhoods preserve swarm diversity by hindering the flow of information through the network [15]. This move prevents the particles from reaching the global best particle immediately or getting trap in a local optimum but allows them to explore larger search space [11, 15]. This beneficial element inspired the existence of $F_{RB}$ which is functioning as neighbourhoods best solution-ABSA version. In addition, $F_{RB}$ also forms the main link between $F_{LB}$ and $F_{GB}$ values. So $F_{RB}$ acts as a leverage instrument to balance finely between exploration (diversification) and exploitation (intensification) processes of the algorithm and so to help the algorithm escape from premature convergence.

The initialisation of these levels will help the ABSA to refine the search for the best solution by a colony of bats in the search space in each step and leave out bad solutions immediately. As a result, the algorithm takes less time to converge to the optimum solution. In point of fact, [9] mentioned that many types of research show that communication between individuals within a group is important where the overall performance of the group is affected by the structure of the social network. Besides, [11] argued that the distribution of information via distant acquaintances is crucial, such that it possesses information that a colleague might not. In conjunction to that, the four levels of the best solution created in ABSA ideally match with the information transfer mechanisms practised by a colony of bats as explored by [3]. These are intentional signalling match to $F_{SP}$, local enhancement match to $F_{LB}$, social facilitation match to $F_{RB}$ and imitative learning match to $F_{GB}$.

The reciprocal altruism characteristic has further been incorporated into ABSA to strengthen the procedure of colony searching for the best solution. This reciprocal altruism behaviour widely runs through a colony of bats as reported by many researchers in bats ecology [3, 5, 24]. By inserting this behaviour into the algorithm, a member of the colony will disseminate and share the location of the best fitness found so far to other bats. As a result, all bats will fly to the best prey ever found when the search process comes to an end. The adoption of this real prey hunting behaviour of the colony of bats into the algorithm is symbolised by two levels of arithmetic mean.

For every bat, the arithmetic mean evaluates the balancing point between $pos_{SP}$, $pos_{LB}$ and $pos_{RB}$ in current iteration (*t*) with $pos_{GB}$ of the latest $F_{GB}$ to be appoint as a new $pos_{SP}$ for next iteration (*t*+1). The first level of arithmetic mean involves measuring of central tendency between $pos_{SP}$, $pos_{LB}$ and $pos_{RB}$ of each bat for current iteration only. Next, the second level of arithmetic mean finds the central tendency between the position value resulted from the first level of arithmetic mean and $pos_{GB}$. As a result, during new iteration, every bat will start to transmit a set of new beams from the $pos_{SP}$ which has been specified after considering (or sharing) the balancing point of the positions of all four level of best fitness solutions; $F_{SP}$, $F_{LB}$, $F_{RB}$ and $F_{GB}$. The two levels of arithmetic mean is expressed as Eq. 14 follows:

14 $$\begin{aligned} pos_{SP}(t+1) = \frac{\frac{pos_{SP}(t)+pos_{LB}(t)+pos_{RB}(t)}{3}+pos_{GB}}{2} \end{aligned}$$

Based on these modifications, the basic steps of the ABSA are represented as the pseudo code in Algorithm 3.

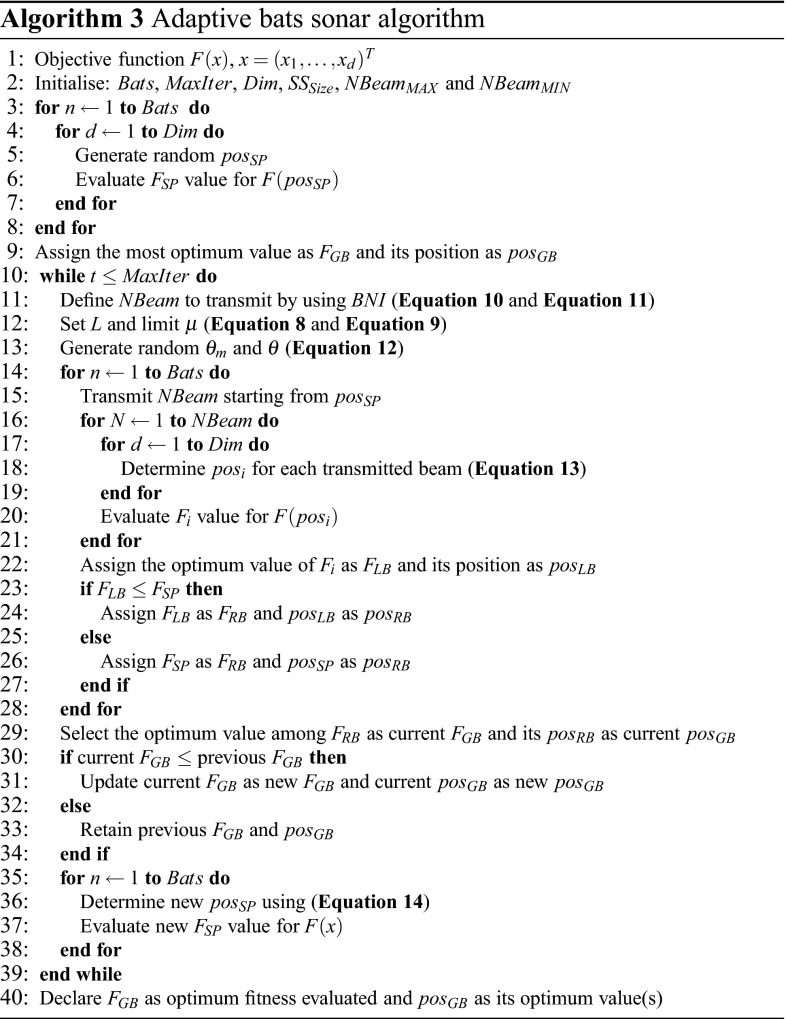



## Effects of *number of bats* and *number of iterations* to the performances of ABSA

Any swarm intelligence algorithm requires setting the values of several algorithm parameters correctly because these parameter values have a significant impact on the performance and efficiency of the algorithm [19]. The size of population and number of iterations used are the main parameters in most of the swarm intelligence algorithms. In BSA and ABSA algorithms, the size of a population is referred to the *number of bats* (*Bats*). However, BSA by [21] applied three bats only while in ABSA the number of bats used are between 700 and 1000 bats, as motivated by the study reported by [17] and [22].

**Fig. 3:**
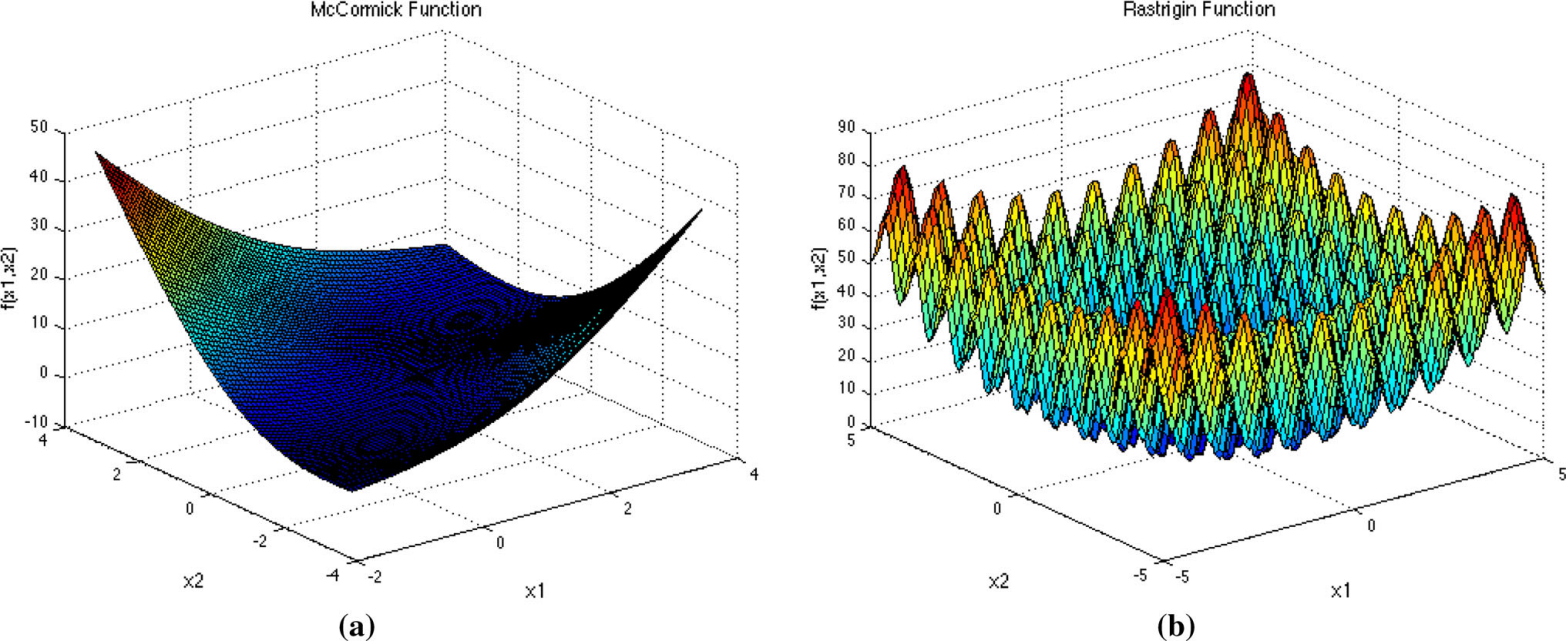
Functions used to evaluate the effects of *Bats* and *MaxIter* on the performances of BSA and ABSA. **a** McCormick function. **b** Rastrigin function

On the other hand, the *number of iterations* (*MaxIter*) used in both algorithms has been set to 100. This value is favourably enough for the bats to explore fully the search space for the best prey (best fitness value). The chosen value is twice the maximum of what *MaxIter* used in PSO when the algorithm was first introduced in 1995 [10]. The overall performance of ABSA is better than BSA not because of the large difference *Bats* used at various number of iterations only, but due to the improvement and modifications made to the original BSA. To demonstrate this, both BSA and ABSA are tested with two different benchmark functions as follows:

- McCormick function This function as in Fig. 3a is unimodal test function and is defined as Eq. 15:
  15 $$\begin{aligned} F(x)=\sin (x_{1}+x_{2})+(x_{1}-x_{2})^2-1.5x_{1}+2.5 x_{2}+1 \end{aligned}$$
  where
  $$\begin{aligned}&x_{1}\in [-1.5,4.0] \\&\quad x_{2}\in [-3.0,4.0] \end{aligned}$$
  The global minimum is $F(x^{*})=-1.9132$ at $x^{*}=(-0.54719,-1.54719)$.
- Rastrigin function This function is a multimodal test function with several regularly distributed local minimum. This function as plot in Fig. 3b is defined as Eq. 16:
  16 $$\begin{aligned} F(x) = 10d+\sum \limits _{i=1}^{d}\left[ x_{i}^2-10\cos (2\pi x_{i})\right] \end{aligned}$$
  where
  $$\begin{aligned} x_{i}\in [-5.12,5.12],\,\,i=1,\ldots ,N \end{aligned}$$
  The global minimum at $F(x^{*})=0$ at $x^{*}=(0,\ldots ,0).$ The test of this function used $d=3$.

In both cases, the number of *Bats* used were 3, 100 and 700 while the *MaxIter* is fixed to 25 and 100. So, number of function evaluations (*NFE*s) defined as Eq. 17:

17 $$\begin{aligned} NFE = Bats \times MaxIter \end{aligned}$$

for each BSA and ABSA are 75, 300, 2500, 10,000, 17,500 and 70,000.

**Table 1:** Best global optimum value achieved by BSA and ABSA for McCormick function with different *Bats* over different *MaxIter*

*Bats*	*MaxIter*	Optimum value of *F*(*x*)	BSA	ABSA	$NFE\hbox {s}$
3	25	−1.9132	−1.8464	−1.9132	75
	100		−1.9130	−1.9127	300
100	25		−1.9130	−1.9132	2500
	100		−1.9123	−1.9132	10,000
700	25		−1.9126	−1.9132	17,500
	100		−1.9132	−1.9132	70,000

**Table 2:** Best global optimum value achieved by BSA and ABSA for Rastrigin function with different *Bats* over different *MaxIter*

*Bats*	*MaxIter*	Optimum value of *F*(*x*)	BSA	ABSA	$NFE\hbox {s}$
3	25	0.0000	3.6481	0.7116	75
	100		1.2568	1.2740E−1	300
100	25		0.9951	3.8270E−6	2500
	100		5.1865E−1	5.8799E−7	10,000
700	25		2.1431E−1	3.2585E−8	17,500
	100		7.0612E−2	4.9231E−10	70,000

**Fig. 4:**
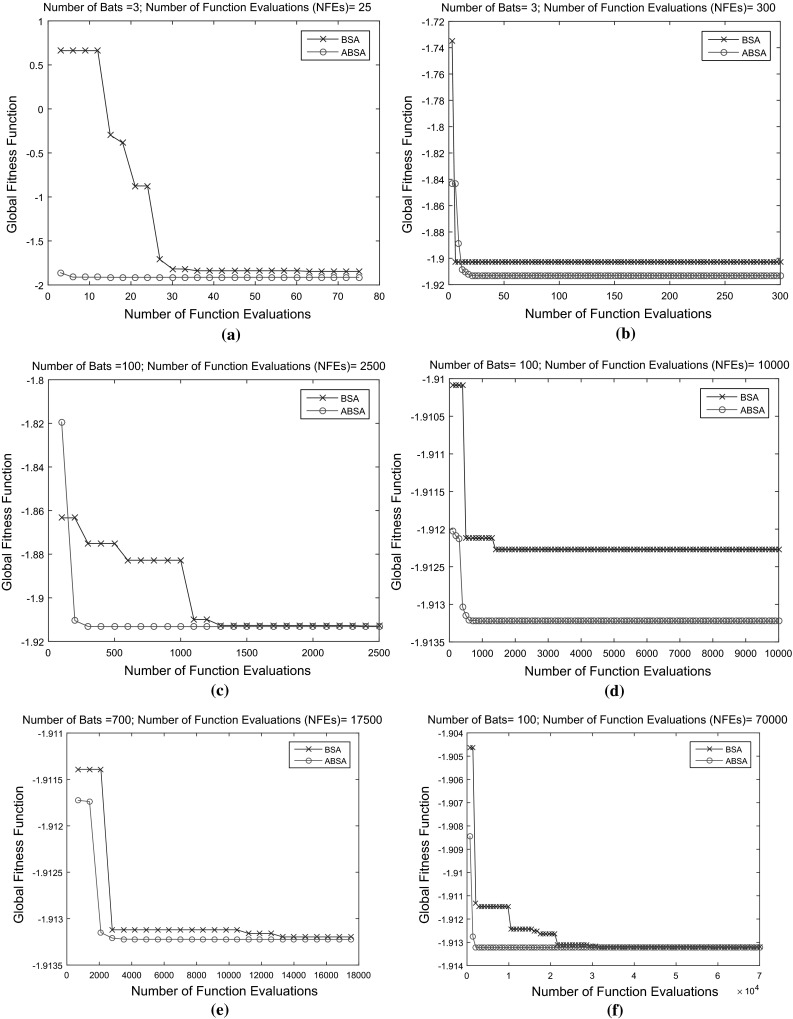
McCormick functions: comparison of performance of the original BSA and the developed ABSA. **a** 3 bats and 25 iterations. **b** 3 bats and 100 iterations. **c** 100 bats and 25 iterations. **d** 100 bats and 100 iterations. **e** 700 bats and 25 iterations. **f** 700 bats and 100 iterations

Table 1 and Fig. 4 depict the best results obtained by the BSA and ABSA in optimising the McCormick function. It is noted that the developed ABSA outperformed the original BSA at various *Bats* used with different *MaxIter* to accelerate the convergence rate to accurate known global optimum.

**Fig. 5:**
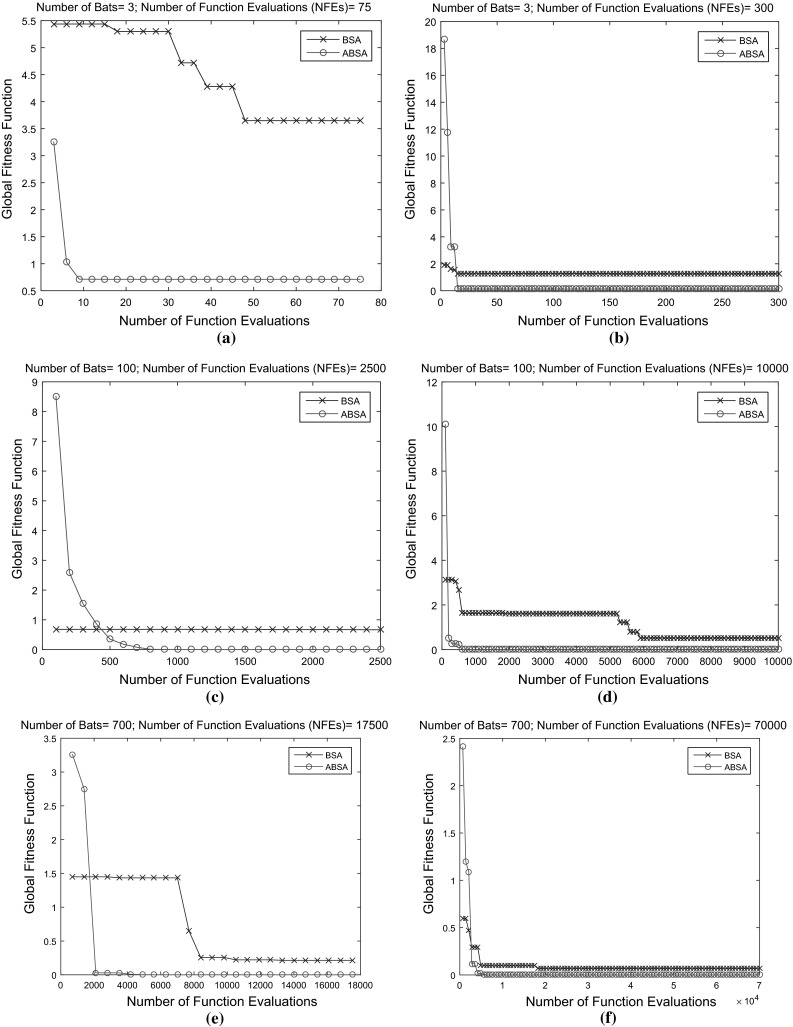
Rastrigin functions: comparison of performance of the original BSA and the developed ABSA. **a** 3 bats and 25 iterations. **b** 3 bats and 100 iterations. **c** 100 bats and 25 iterations. **d** 100 bats and 100 iterations. **e** 700 bats and 25 iterations. **f** 700 bats and 100 iterations

As evident from Table 2 and Fig. 5, ABSA further showed promising results as compared to the original BSA method. The obtained results in optimising the Rastrigin function suggested that the ABSA succeeded to converge faster and near accurate to the best known global optimum at various numbers of bats used with different numbers of iterations as compared to original BSA.

At this point, the preliminary conclusion drawn about the ABSA as compared to original BSA is that ABSA has successfully converged faster with better accuracy to the known global optimum when compared with BSA without it being affected by a large difference in the number of bats used at various numbers of iterations.

## Performance of adaptive bats sonar algorithm on established single objective optimisation benchmark test functions

There are many benchmark test functions that can be used for testing and validating the developed algorithm. Ten single objective optimisation benchmark test functions, as summarised in Table 3 are used to show the efficiency of ABSA. The first three test functions (FN01, FN02 and FN03) have previously been used by [21] to demonstrate the performance of the original BSA. All the three test functions have maximum values at their optimum. The remaining test functions have minimum values as their optimum [14]. In this validation, the functions FN04, FN05, FN06 and FN07 were run in three different dimensions, namely three dimensions (FN0*a), five dimensions (FN0*b) and ten dimensions (FN0*c).

Two other algorithms are also tested on the same 10 test functions as in Table 3 to verify the performance of ABSA on a comparative basis. The algorithms are bats sonar algorithm (BSA) by [21] and bat algorithm (BA) by [27]. The parameters used for the BSA are the same as originally used by [21]. These were three bats, five beams (*N*) in each transmitted signal and the angle between any two successive beams was fixed at $\pi \setminus 12$. Similarly, the standard algorithm parameters are used with BA. These were population size of 50, pulse rate (*r*) equal to 0.5, loudness (*A*) fixed at 0.25 and random number less than 1 for beta ($\beta$).

Each algorithm was run 30 times to allow it to carry out meaningful statistical analysis. The maximum number of iterations for each run was set to 100. All three algorithms on the ten function evaluations obtained the result of *best*, *mean*, *worst* and *standard deviation* values. To evaluate the statistical significance of the ABSA, one-way analysis of variance (ANOVA) with post-test (Dunnett’s test type) was applied, and the null hypothesis was rejected at the confidence level of 5 %.

**Table 3:** Benchmark functions used to validate the performance of ABSA

Label	Function name (type)	Function	Optimum value of *F*(*x*)	Range of solution space
FN01	Third-order polynomial with a single variable (Max)	$F(x)=x^{3}-5x^{2}-20x$	15.4564	$-65.12\le x\le 65.12$
FN02	Polynomial with two variables (Max)	$F(x_{1},x_{2})=x_{1}^{3}-5x_{1}^{2}-2.04x_{2}^{2}+4x_{2}$	1.9608	$-3\le (x_{1},x_{2})\le 3$
FN03	Exponential with two variables (Max)	$F(x_{1},x_{2})=x_{1}\exp ^{(-x_{1}^{2}-x_{2}^{2})}$	0.4289	$-2\le (x_{1},x_{2})\le 2$
FN04	De Jong’s (Min)	$F(x)=\sum \nolimits _{i=1}^{n} x_{i}^{2}$	0.0000	$-5.12\le x_{i}\le 5.12,\,\,i=1,\ldots ,N$
FN05	Weighted sphere model (Min)	$F(x)=\sum \nolimits _{i=1}^{n}(i\cdot x_{i}^{2})$	0.0000	$-5.12\le x_{i}\le 5.12,\,\, i=1,\ldots ,N$
FN06	Shwefel’s (Min)	$F(x)=\sum \nolimits _{i=1}^{n}(i\cdot x_{i}^{2})$	0.0000	$-65.536\le x_{i} \le 65.536,\,\, i=1,\ldots ,N$
FN07	Rosenbrock’s valley (Min)	$F(x)= \sum \nolimits _{i=1}^{n}[100(x_{i+1}-x_{i}^{2})^{2})+(1-x_{i})^{2}]$	0.0000	$-2.048\le x_{i}\le 2.048,\,\, i=1,\ldots ,N$
FN08	Easom’s (Min)	$F(x_{1},x_{2})=-\cos x_{1}\cos x_{2}\exp (-(x_{1}-\pi )^{2}-(x_{2}-\pi )^{2})$	−1.0000	$-100\le (x_{1},x_{2})\le 100$
FN09	Goldstein-Price’s (Min)	$F(x_{1},x_{2})=(1+(x_{1}+x_{2}+1)^{2} (19-14x_{1}+3x_{1}^{2}-14x_{2}+6x_{1} x_{2}+3x_{2}^{2} ))(30+(2x_{1}-3x_{2})^{2} (18-32x_{1}+12x_{1}^{2}+48x_{2}-36x_{1} x_{2}+27x_{2}^{2}))$	3.0000	$-2\le (x_{1},x_{2})\le 2$
FN10	Booth’s (Min)	$F(x_{1},x_{2})=(x_{1}+2x_{2}-7)^{2}+(2x_{1}+x_{2}-5)^{2}$	0.0000	$-10\le (x_{1},x_{2})\le 10$

**Fig. 6:**
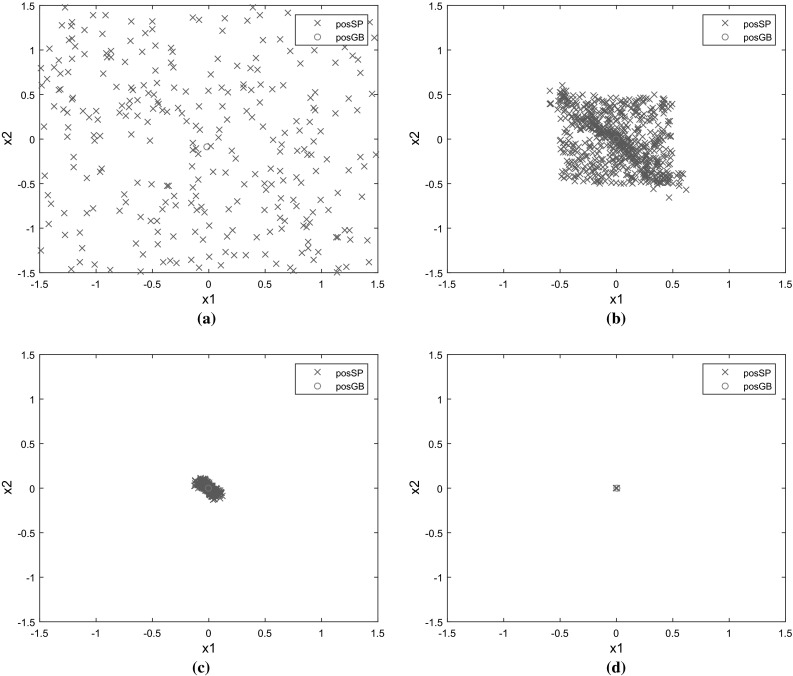
Locations of 1000 bats using ABSA for 2 dimensional De Jong function. **a** Iteration 1. **b** Iteration 5. **c** Iteration 20. **d** Iteration 50

Figure 6a–d shows the search patterns of 1000 bats positions using ABSA for 2 dimension De Jong function. Its global minimum $F(x)=0$ was obtainable for $x_{i}=0$, $i=1,\ldots ,N$. In iteration 1, 1000 bats scattered at various locations in the designated search space. Bats started to converge to the final value of $x_{i}$ as the iteration increased. At iteration 50, all 1000 bats settled to the optimum values of $x_{1}=0$ and $x_{2}=0$.

The results of the computer simulations for ABSA algorithm are given in Table 4. As noted, the algorithm achieved the global optimum value with zero or very small *standard deviation*. Comparative results of the *best*, *worst* and *mean* solutions with *standard deviation* values of the investigated algorithms are shown in Tables 5, 6, 7 and 8 respectively.

**Table 4:** Statistical results obtained for ABSA with 10 test functions of different dimensions over 30 independent runs of 100 iterations each

Function number	Dim	Optimum *F*(*x*)	*Best*	*Mean*	*Worst*	*SD*
FN01	1	15.4564	15.4564	15.4564	15.4564	0.0000
FN02	2	1.9608	1.9608	1.9608	1.9608	0.0000
FN03	2	0.4289	0.4289	0.4289	0.4289	0.0000
FN04a	3	0.0000	2.2810E−13	1.2374E−9	9.6814E−9	2.4540E−9
FN04b	5	0.0000	1.2726E−11	2.1789E−8	2.3951E−7	5.2963E−8
FN04c	10	0.0000	1.3720E−4	5.4975E−2	3.9510E−1	1.0842E−1
FN05a	3	0.0000	4.8111E−12	4.0332E−10	1.5621E−9	4.5575E−10
FN05b	5	0.0000	4.4514E−11	1.1890E−8	6.3666E−8	1.5027E−8
FN05c	10	0.0000	2.6957E−4	2.5186E−2	6.6100E−2	1.7923E−2
FN06a	3	0.0000	1.1643E−11	2.0870E−9	7.3697E−9	2.1982E−9
FN06b	5	0.0000	5.2555E−10	5.4807E−8	4.2394E−7	1.0912E−7
FN06c	10	0.0000	6.2212E−5	5.6951E−3	2.3500E−2	7.7790E−3
FN07a	3	0.0000	1.8990E−12	2.9536E−9	1.8916E−8	4.3566E−9
FN07b	5	0.0000	3.3335E−11	1.6080E−7	4.6234E−6	8.4319E−7
FN07c	10	0.0000	2.3001E−12	3.9551E−9	3.0717E−8	7.6405E−9
FN08	2	−1.0000	−1.0000	−1.0000	−1.0000	0.0000
FN09	2	3.0000	3.0000	3.0000	3.0000	0.0000
FN10	2	0.0000	0.0000	0.0000	0.0000	0.0000

**Table 5:** The *best* solution obtained by BA, BSA and ABSA with 10 test functions of different dimensions over 30 independent runs of 100 iterations each

Function number	Dim	Optimum *F*(*x*)	BA	BSA	ABSA
FN01	1	15.4564	15.4564	15.4564	15.4564
FN02	2	1.9608	1.9832	1.9606	1.9608
FN03	2	0.4289	0.4280	0.4289	0.4289
FN04a	3	0.0000	1.1985E−7	1.8211E−5	2.2810E−13
FN04b	5	0.0000	1.0854E−6	3.9700E−2	1.2726E−11
FN04c	10	0.0000	1.2000E−3	8.0770E−1	1.3720E−4
FN05a	3	0.0000	2.5850E−7	1.4324E−9	4.8111E−12
FN05b	5	0.0000	1.1000E−3	5.7284E−5	4.4514E−11
FN05c	10	0.0000	4.6000E−3	8.6000E−3	2.6957E−4
FN06a	3	0.0000	7.5661E−8	1.7246E−9	1.1643E−11
FN06b	5	0.0000	1.0000E−3	3.3504E−4	5.2555E−10
FN06c	10	0.0000	2.3800E−2	4.5000E−3	6.2212E−5
FN07a	3	0.0000	3.4954E−9	3.5720E−7	1.8990E−12
FN07b	5	0.0000	2.1000E−3	1.3993E−4	3.3335E−11
FN07c	10	0.0000	8.6000E−3	2.7000E−3	2.3001E−12
FN08	2	−1.0000	−1.0000	−0.9999	−1.0000
FN09	2	3.0000	3.0000	3.0060	3.0000
FN10	2	0.0000	0.0000	0.0001	0.0000

**Table 6:** The *worst* solution obtained by BA, BSA and ABSA with 10 test functions of different dimensions over 30 independent runs of 100 iterations each

Function number	Dim	Optimum *F*(*x*)	BA	BSA	ABSA
FN01	1	15.4564	15.3302	15.4175	15.4564
FN02	2	1.9608	1.9006	1.9032	1.9608
FN03	2	0.4289	0.4024	0.4221	0.4289
FN04a	3	0.0000	9.8722E−5	8.5000E−3	9.6814E−9
FN04b	5	0.0000	6.7300E−2	6.9350E−1	2.3951E−7
FN04c	10	0.0000	1.1070E−1	1.8506	3.9510E−1
FN05a	3	0.0000	8.6962E−4	1.4619E−5	1.5621E−9
FN05b	5	0.0000	5.1300E−2	9.5000E−3	6.3666E−8
FN05c	10	0.0000	8.8270E−1	9.8190E−1	6.6100E−2
FN06a	3	0.0000	8.2515E−4	3.9698E−5	7.3697E−9
FN06b	5	0.0000	8.9700E−2	9.4000E−2	4.2394E−7
FN06c	10	0.0000	4.9420E−1	9.0690E−1	2.3500E−2
FN07a	3	0.0000	9.4882E−4	8.5589E−4	1.8916E−8
FN07b	5	0.0000	9.9000E−2	1.4600E−2	4.6234E−6
FN07c	10	0.0000	8.7030E−1	9.3110E−1	3.0717E−8
FN08	2	−1.0000	−1.4070	−0.8110	−1.0000
FN09	2	3.0000	3.4618	3.8640	3.0000
FN10	2	0.0000	0.3314	0.1215	0.0000

**Table 7:** The *mean* solution obtained by BA, BSA and ABSA with 10 test functions of different dimensions over 30 independent runs of 100 iterations each

Function number	Dim	Optimum *F*(*x*)	BA	BSA	ABSA
FN01	1	15.4564	15.4458	15.4438	15.4564
FN02	2	1.9608	1.9308	1.9401	1.9608
FN03	2	0.4289	0.4177	0.4262	0.4289
FN04a	3	0.0000	3.6929E−5	2.6683E−3	1.2374E−9
FN04b	5	0.0000	5.1481E−3	4.1950E−1	2.1789E−8
FN04c	10	0.0000	2.6150E−2	1.4665	5.4975E−2
FN05a	3	0.0000	8.0776E−5	1.1634E−6	4.0332E−10
FN05b	5	0.0000	1.4917E−2	3.6329E−3	1.1890E−8
FN05c	10	0.0000	3.4812E−1	4.1136E−1	2.5186E−2
FN06a	3	0.0000	8.6964E−5	3.2073E−6	2.08470E−9
FN06b	5	0.0000	2.4963E−2	3.0683E−2	5.4807E−8
FN06c	10	0.0000	1.5900E−1	3.4829E−1	5.6951E−3
FN07a	3	0.0000	5.9211E−4	3.7671E−4	2.9536E−9
FN07b	5	0.0000	3.5097E−2	4.5607E−3	1.6080E−7
FN07c	10	0.0000	3.9344E−1	1.9216E−1	3.9551E−9
FN08	2	−1.0000	−1.2144	−0.9554	−1.0000
FN09	2	3.0000	3.0938	3.3215	3.0000
FN10	2	0.0000	0.0869	0.0331	0.0000

**Table 8:** The *standard deviation* obtained by BA, BSA and ABSA with 10 test functions of different dimensions over 30 independent runs of 100 iterations each

Function number	Dim	Optimum *F*(*x*)	BA	BSA	ABSA
FN01	1	15.4564	0.0278	0.0095	0.0000
FN02	2	1.9608	0.0188	0.0184	0.0000
FN03	2	0.4289	0.0081	0.0025	0.0000
FN04a	3	0.0000	3.2411E−5	2.3319E−3	2.4540E−9
FN04b	5	0.0000	1.2468E−2	1.7864E−1	5.2963E−8
FN04c	10	0.0000	2.4978E−2	3.3193E−1	1.0842E−1
FN05a	3	0.0000	1.9681E−4	2.7481E−6	4.5575E−10
FN05b	5	0.0000	1.2349E−2	3.0154E−3	1.5027E−8
FN05c	10	0.0000	2.5533E−1	3.0597E−1	1.7923E−2
FN06a	3	0.0000	1.9133E−4	8.3095E−6	2.1982E−9
FN06b	5	0.0000	1.8628E−2	3.4283E−2	1.0912E−7
FN06c	10	0.0000	1.0826E−1	2.5159E−1	7.7790E−3
FN07a	3	0.0000	2.5279E−4	2.8526E−4	4.3566E−9
FN07b	5	0.0000	3.5821E−2	4.2380E−3	8.4319E−7
FN07c	10	0.0000	2.7202E−1	2.7346E−1	7.6405E−9
FN08	2	−1.0000	0.1308	0.0438	0.0000
FN09	2	3.0000	0.2003	0.3021	0.0000
FN10	2	0.0000	0.0818	0.0356	0.0000

As seen in Table 5, the ABSA approach found the exact or close global optimum value of thirteen out of the eighteen functions (FN02, FN04a-c, FN05a-c, FN06a-c and FN07a-c) through 30 runs. From one function (FN01), ABSA produced results similar to both BA and BSA. Moreover, ABSA achieved similar *best* value with BSA on FN03, with BA in three functions, namely FN08, FN09 and FN10. Overall, as noted, the ABSA *best* results were superior to those achieved with BSA and BA.

As noted in the *worst* solution results given in Table 6, ABSA outperformed BA and BSA in all eighteen functions tested. Even for the *worst* results, ABSA successfully achieved accurate or very near accurate results to global optimum points. Similarly, on the *mean* solutions as shown in Table 7, ABSA achieved accurate performance as compared to BA and BSA for seventeen out of the eighteen function evaluations. Even though for the FN04c the BA achieved better optimum solution compared to ABSA, the gap between them was small.

As far as *standard deviation* is concerned, the results in Table 8 show the best precision exhibited by ABSA. Less variation (some functions, no variation) of optimum solution from the *mean* values was produced by implementing ABSA on all test functions except FN04c. For FN04c, BA was able to achieve smaller *standard deviation* value compared to that achieved with ABSA but the difference was not significant.

Table 9 shows a comparison of the performance of ABSA with BA and BSA using one-way analysis of variance (ANOVA) on the *mean* value $\pm$*standard deviation* of the global optimum. It is noted that at 95 % confident interval, ABSA was statistically significant to achieve better global optimum solution ahead of BA and BSA. Overall, it can be concluded that ABSA outperforms BA and BSA for accuracy and precision to search for a global optimum solution either in maximisation or minimisation problems.

**Table 9:** Performance comparison using one-way analysis of variance (ANOVA) between BA, BSA and ABSA with 10 test functions of different dimensions over 30 independent runs of 100 iterations each

FN No.	BA	BSA	ABSA	Significantly
FN01	15.4564 ± 0.0278	15.4538 ± 0.0095	15.4564 ± 0.0000	Yes
FN02	1.9308 ± 0.0188	1.9401 ± 0.0184	1.9608 ± 0.0000	Yes
FN03	0.4177 ± 0.0081	0.4262 ± 0.0025	0.4289 ± 0.0000	Yes
FN04a	3.6929E−5 ± 3.2411E−5	2.6683E−3 ± 2.3319E−3	1.2374E−9 ± 2.4540E−9	Yes
FN04b	5.1481E−3 ± 1.2468E−2	4.1950E−1 ± 1.7864E−1	2.1789E−8 ± 5.2963E−8	Yes
FN04c	2.6150E−2 ± 2.4978E−2	1.4665 ± 3.3193E−1	5.4975E−2 ± 1.0842E−1	Yes
FN05a	8.0776E−5 ± 1.9681E−4	1.1634E−6 ± 2.7481E−6	4.0332E−10 ± 4.5575E−10	Yes
FN05b	1.4917E−2 ± 1.2349E−2	3.6329E−3 ± 3.0154E−3	1.1890E−8 ± 1.5027E−8	Yes
FN05c	3.4812E−1 ± 2.5533E−1	4.1136E−1 ± 3.0597E−1	2.5186E−2 ± 1.7923E−2	Yes
FN06a	8.6964E−5 ± 1.9133E−4	3.2073E−6 ± 8.3095E−6	2.0870E−9 ± 2.1982E−9	Yes
FN06b	2.4963E−2 ± 1.8628E−2	3.0683E−2 ± 3.4283E−2	5.4807E−8 ± 1.0912E−7	Yes
FN06c	1.5900E− ± 1.0826E−1	3.4829E−1 ± 2.5159E−1	5.6951E−3 ± 7.7790E−3	Yes
FN07a	5.9211E−4 ± 2.5279E−4	3.7671E−4 ± 2.8526E−4	2.9536E−9 ± 4.3566E−9	*Yes*
FN07b	3.5097E−2 ± 3.5821E−2	4.5607E−3 ± 4.2380E−3	1.6080E−7 ± 8.4319E−7	Yes
FN07c	3.9344E−1 ± 2.7202E−1	1.9216E−1 ± 2.7346E−1	3.9551E−9 ± 7.6405E−9	Yes
FN08	−1.2144 ± 0.1308	−0.9554 ± 0.0438	−1.0000 ± 0.0000	Yes
FN09	3.0938 ± 0.2003	3.3215 ± 0.3021	3.0000 ± 0.0000	Yes
FN10	0.0869 ± 0.0818	0.0331 ± 0.0356	0.0000 ± 0.0000	Yes

**Fig. 7:**
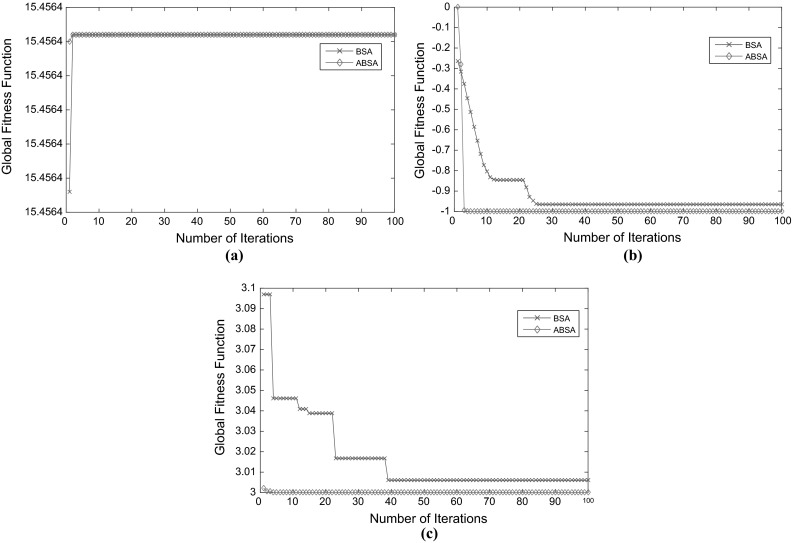
Convergence to global best fitness function achieved by ABSA and BSA for selected test functions. **a** Third-order polynomial with single variable. **b** Easom’s function. **c** Goldstein–Price’s function

Figure 7 shows convergence to global best fitness function value achieved by the ABSA as compared to BSA for selected single objective optimisation benchmark test functions. However, these do not account for differing computational costs, as in reality, ABSA has taken longer time than BSA to arrive at a maximum number of iteration. This is due to the new structure and additional steps incorporated into the original BSA to arrive at the developed ABSA. The graphical results show that ABSA was able to converge to global best fitness for each function in a smaller number of iterations compared to BSA. Moreover, with several random approaches introduced to locate the starting positions in ABSA, the algorithm is potentially able to start the search process at locations close to the optimum point and promptly move to the absolute global best point.

**Fig. 8:**
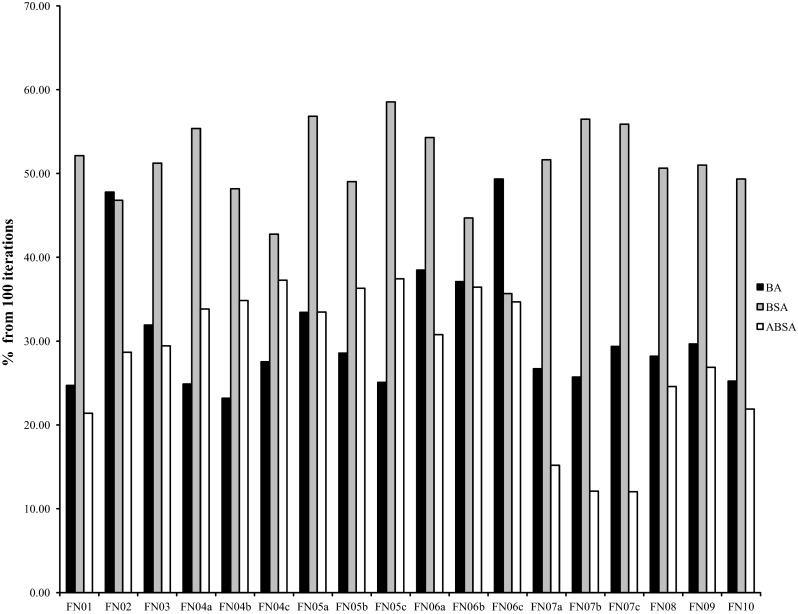
Comparison of average number of iterations to achieve global optimum solution

Table 10 presents the results of one-way analysis of variance (ANOVA) on the *mean* iteration value $\pm$*standard deviation* of iteration number to arrive at a global optimum solution. The results show that at the 95 % confident interval, ABSA significantly performed better than BA and BSA to converge to the global optimum solution faster. According to Fig. 8, on average, in 100 iterations, the ABSA needed around 12–37 % iterations to reach the global optimum solution. The algorithm outperformed BA and BSA, which took 24–49 and 35–58 % iterations respectively. This implies that ABSA has faster convergence ability to a global optimum solution either for maximisation or minimisation problems as compared to BA and BSA.

**Table 10:** Performance comparison in terms of faster convergence to global optimum in 100 iterations using one-way analysis of variance (ANOVA) between BA, BSA and ABSA with 10 test functions of different dimensions over 30 independent runs

FN No.	BA	BSA	ABSA	Significantly
FN01	24.70 ± 15.12	52.13 ± 29.63	21.40 ± 8.79	Yes
FN02	47.77 ± 2.60	46.80 ± 29.51	28.67 ± 13.50	Yes
FN03	31.93 ± 12.60	51.23 ± 34.23	29.43 ± 13.88	Yes
FN04a	24.87 ± 16.87	55.37 ± 29.05	33.83 ± 11.11	Yes
FN04b	23.17 ± 13.98	48.17 ± 31.09	34.83 ± 11.11	Yes
FN04c	27.53 ± 14.49	42.77 ± 30.03	37.27 ± 8.79	Yes
FN05a	33.43 ± 10.25	56.83 ± 30.30	33.47 ± 11.75	Yes
FN05b	28.57 ± 15.93	49.03 ± 32.18	36.30 ± 9.55	Yes
FN05c	25.07 ± 12.65	58.53 ± 35.15	37.43 ± 9.26	Yes
FN06a	38.47 ± 9.78	54.30 ± 28.75	30.77 ± 12.14	Yes
FN06b	37.10 ± 7.44	44.70 ± 30.50	36.43 ± 10.81	Yes
FN06c	49.33 ± 7.37	35.67 ± 29.38	34.67 ± 11.56	Yes
FN07a	26.70 ± 15.62	51.63 ± 27.50	15.17 ± 10.02	Yes
FN07b	25.70 ± 11.76	56.47 ± 29.83	12.10 ± 5.84	Yes
FN07c	29.37 ± 11.94	55.87 ± 28.33	12.03 ± 3.37	Yes
FN08	28.20 ± 13.65	50.63 ± 29.89	24.57 ± 14.07	Yes
FN09	29.67 ± 16.58	51.00 ± 27.67	26.87 ± 14.21	Yes
FN10	25.23 ± 15.02	49.33 ± 26.75	21.90 ± 14.39	Yes

## Conclusion

With the aim of improving accuracy, precision and convergence rate of the original bats sonar algorithm (BSA), an improved algorithm by altering and incorporating new characteristics into the algorithm has been proposed. This is referred to as an adaptive bats sonar algorithm (ABSA). This includes modification of the number of bats, number of beams and their lengths, starting angle and introduction of new techniques comprising beam number increment (BNI), four levels of best solution and reciprocal altruism behaviour of real bats. Numerical simulations with single objective optimisation benchmark test functions have demonstrated the efficiency of the ABSA toward the stated aims and its superior performance in comparison to BSA and bat algorithm (BA). Future work will look at application and assessment of performance of the ABSA in engineering problems and in comparison to other algorithms. Moreover, the extension of the algorithm to solve constrained optimisation problems as well as multi objective optimisation problems will be considered later.
